# Hepatic SMARCA4 predicts HCC recurrence and promotes tumour cell proliferation by regulating SMAD6 expression

**DOI:** 10.1038/s41419-017-0090-8

**Published:** 2018-01-19

**Authors:** Zhiao Chen, Xinyuan Lu, Deshui Jia, Ying Jing, Di Chen, Qifeng Wang, Fangyu Zhao, Jinjun Li, Ming Yao, Wenming Cong, Xianghuo He

**Affiliations:** 1 Fudan University Shanghai Cancer Center and Institutes of Biomedical Sciences; Department of Oncology, Shanghai Medical College, Fudan University, 200032 Shanghai, China; 2 Department of Pathology, Eastern Hepatobiliary Surgery Hospital, Second Military Medical University, 200438 Shanghai, China; 3 State Key Laboratory of Oncogenes and Related Genes, Shanghai Cancer Institute, Renji Hospital, Shanghai Jiao Tong University School of Medicine, 200032 Shanghai, China

## Abstract

Hepatocellular carcinoma (HCC) is the most common form of liver cancer and is typically diagnosed at advanced stages. Identification and characterisation of genes within amplified and deleted chromosomal loci can provide new insights into the pathogenesis of cancer and lead to new approaches for diagnosis and therapy. In our previous study, we found a recurrent region of copy number amplification at 19p13.2 in hepatocellular carcinoma (HCC). In the present study, we performed integrated copy number analysis and expression profiling at this locus and a putative cancer gene, SMARCA4/BRG1, was uncovered in this region. BRG1 is a part of the large ATP-dependent chromatin remodelling complex SWI/SNF. The function of BRG1 in various cancers is unclear, including its role in HCC tumorigenesis. Here, we found that BRG1 is upregulated in HCC and that its level significantly correlates with cancer progression in HCC patients. Importantly, we also found that nuclear expression of BRG1 predicts early recurrence for HCC patients. Furthermore, we demonstrated that BRG1 promotes HCC cell proliferation in vitro and in vivo. BRG1 was observed not only to facilitate S-phase entry but also to attenuate cell apoptosis. Finally, we discovered that one of the mechanisms by which BRG1 promotes cell proliferation is the upregulation of SMAD6. These findings highlight the important role of BRG1 in the regulation of HCC proliferation and provide valuable information for cancer prognosis and treatment.

## Introduction

Primary liver cancer is the sixth most common cancer worldwide and is a leading cause of death in Asia^1,2^. Hepatocellular carcinoma (HCC) is the most common form of liver cancer and is typically diagnosed at advanced stages^3^. Identification and characterisation of genes within amplified and deleted chromosomal loci can provide new insights into the pathogenesis of cancer and lead to new approaches for diagnosis and therapy^4^. To identify novel cancer-related genes, we previously identified 1241 loci with somatic copy number alterations (CNAs) in human HCC using Affymetrix genome-wide SNP 6.0 arrays. Importantly, several new CNAs were found, including a recurrent region of copy number amplification at 19p13.2 in HCC^5^. To identify the potential driver genes located in this region, here we performed integrated copy number analysis and expression profiling at this locus, and found that one candidate gene, SMARCA4 (also known as BRG1), was upregulated.

BRG1 is a member of the SWI/SNF family of proteins. Members of this family have helicase and ATPase activities and are thought to regulate the transcription of certain genes by altering the chromatin structure around those genes^6,7^. Growing evidence indicates that these complexes have a widespread role in tumour suppression, because inactivating mutations in several SWI/SNF subunits have recently been identified at a high frequency in a variety of cancers^8^. BRG1 is part of the large ATP-dependent chromatin remodelling complex SWI/SNF, which is required for the transcriptional activation of genes normally repressed by chromatin^9,10^.

Clearly, the reduced expression of SWI/SNF subunits may be a driving mechanism in cancer^10^. Expression of the BRG1 subunit is absent in 15–50% of human primary non-small-cell lung cancer (NSCLC) samples, and mutations in BRG1 have been identified in 35% of NSCLC cell lines^11–13^. In mice, Brg1 heterozygosity results in mammary tumours in 10% of mice, and the second Brg1 allele is always retained, indicating that Brg1 haploinsufficiency can drive tumorigenesis^14,15^. Endo et al. reported homozygous deletion of the BRG1 gene in the HCC cell line SNU398, and copy number losses of the BRG1 gene were observed in primary HCC tumours. Conversely, expression of BRG1 mRNA was found to be significantly upregulated in HCC tumours compared to non-tumour counterparts^16^. In the present study, we also found that BRG1 was upregulated in HCC patients. Although deletions and mutations in the BRG1 gene were identified, the role of BRG1 in HCC tumorigenesis remains unclear. It will be interesting to determine whether overexpression of BRG1 is essential for tumour formation in HCC.

## Results

### BRG1 was upregulated in HCC and significantly correlated with cancer progression and recurrence in HCC patients

**Fig. 1:**
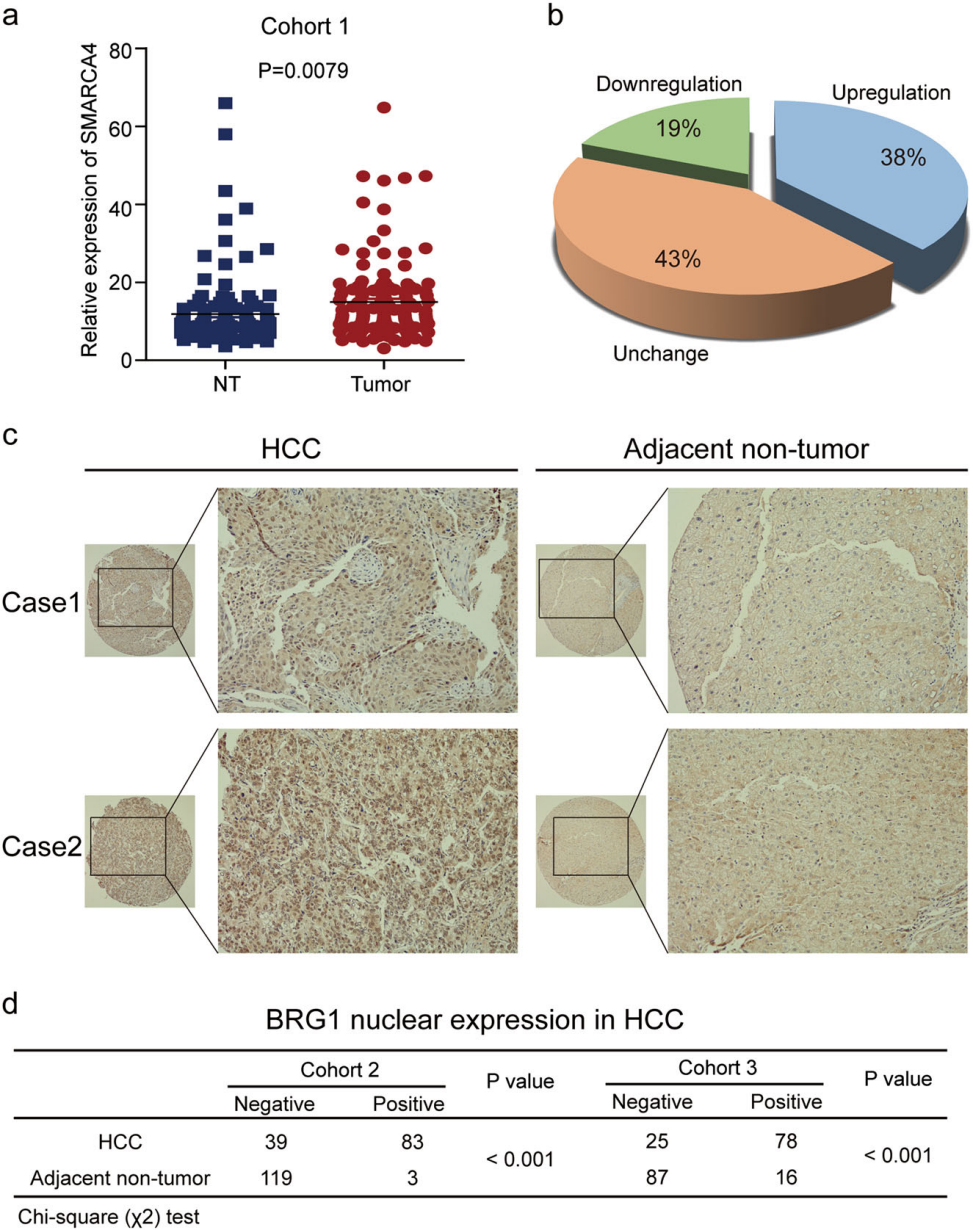
The expression of BRG1 was upregulated in HCC **a** The expression levels of BRG1 were measured using quantitative real-time PCR in 140 tumour and adjacent normal tissues. **b** Significant upregulation of BRG1 in paired HCC/non-tumour samples was defined as a log2-fold change of >1 (i.e., a two-fold change). The pie chart shows the proportion of HCC samples exhibiting upregulation (blue), downregulation (green), and no change (brown). **c** Two representative cases of BRG1 expression in HCC tissues and adjacent non-tumour tissues are shown. Original magnification, 100×. **d** Immunohistochemistry (IHC) analysis of BRG1 in two independent sets of 129 and 118 paired HCC and adjacent non-tumour tissues. The IHC signal intensity in the nucleus was scored as negative or positive. The *P*-values were determined by a *χ*^2^-test

To determine the role of BRG1 in the progression of HCC, we first examined the expression level of BRG1 in 140 paired primary HCC and adjacent non-tumour liver tissues (NTs) using quantitative real-time PCR (cohort 1). The results showed that the mRNA levels of BRG1 were upregulated in HCC compared to NT (Fig. 1a). Moreover, upregulation of BRG1 mRNA was observed in 54 (38%) cases with 100% upregulation as the cut off level (Fig. 1b). In addition, we analysed the expression of BRG1 in HCC, on the basis of curated data generated by The Cancer Genome Atlas-Liver Hepatocellular Carcinoma (TCGA-LIHC) and Oncomine. The results also revealed higher BRG1 expression in HCC tissues than in normal liver tissues in two independent sets of HCC specimens (Supplementary Fig. 1).

Furthermore, we performed a tissue array using immunohistochemical staining of HCC tissues to analyse the protein levels of BRG1 compared to those in matched adjacent NTs. The results showed that BRG1 was primarily localised to the nucleus in the tumour tissue (Fig. 1c). In cohort 2, high BRG1 expression in the nucleus was found in 83 of the 122 (68.0%) primary HCC samples and in 3 of the 122 (2.5%) adjacent NTs (*P* < 0.001). Positive BRG1 expression in cohort 3 was found in 78 of the 103 (75.7%) primary HCC samples and 16 of the 103 (12.6%) adjacent NTs (*P* < 0.001) (Fig. 1d).

**Fig. 2:**
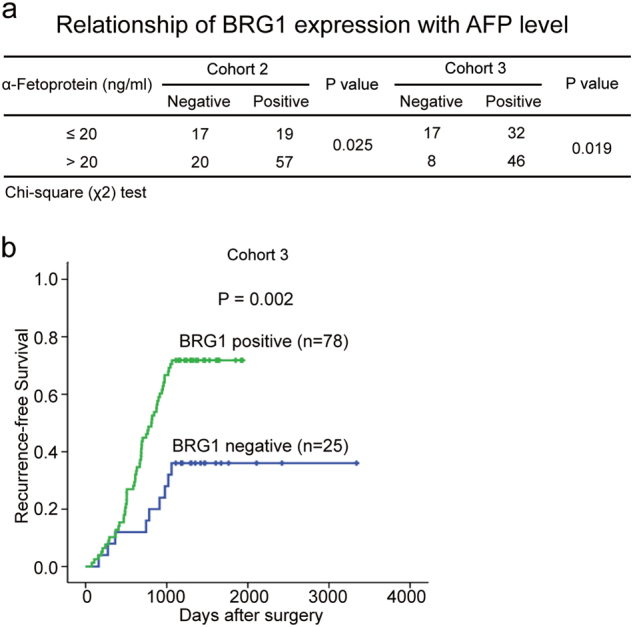
BRG1 expression was significantly correlated with cancer progression and recurrence in HCC patients **a** Correlation of BRG1 expression with serum AFP concentration in patients from two cohorts. **b** Kaplan–Meier analysis of the correlation between BRG1 expression and the time to recurrence of 118 patients with HCC. Log-rank tests were used to determine statistical significance

Because BRG1 expression increased in HCC, we performed further analyses to determine the clinicopathological significance of BRG1 in HCC. BRG1 nuclear staining was observed in 57 of 77 (74.0%) tumour liver tissue from patients whose level of AFP was greater than 20 ng/ml, 19 of 36 (52.8%) tumour liver tissue from patients whose level of AFP was less than or equal to 20 ng/ml in cohort 2 (*P* < 0.05). Importantly, we found that BRG1 overexpression was also correlated with serum AFP concentration in cohort 3 (Fig. 2a). In addition, we evaluated the relationship between BRG1 expression and HCC risk by analysing the tumour liver tissue of early stage HCC patients undergoing curative ablation. However, the expression of BRG1 showed no statistically significant correlation with the overall survival (OS) and recurrence-free survival (RFS) of HCC patients in cohort 2. Importantly, in cohort 3, which included 118 patients who all relapsed a few years later, we found that patients with positive BRG1 expression had a shorter time (less than 3 years) to recurrence (Fig. 2b). Taken together, these data suggest that increased BRG1 expression was a frequent event in human HCC tissues and may be involved in HCC progression.

### BRG1 promotes cell proliferation in vitro and in vivo

**Fig. 3:**
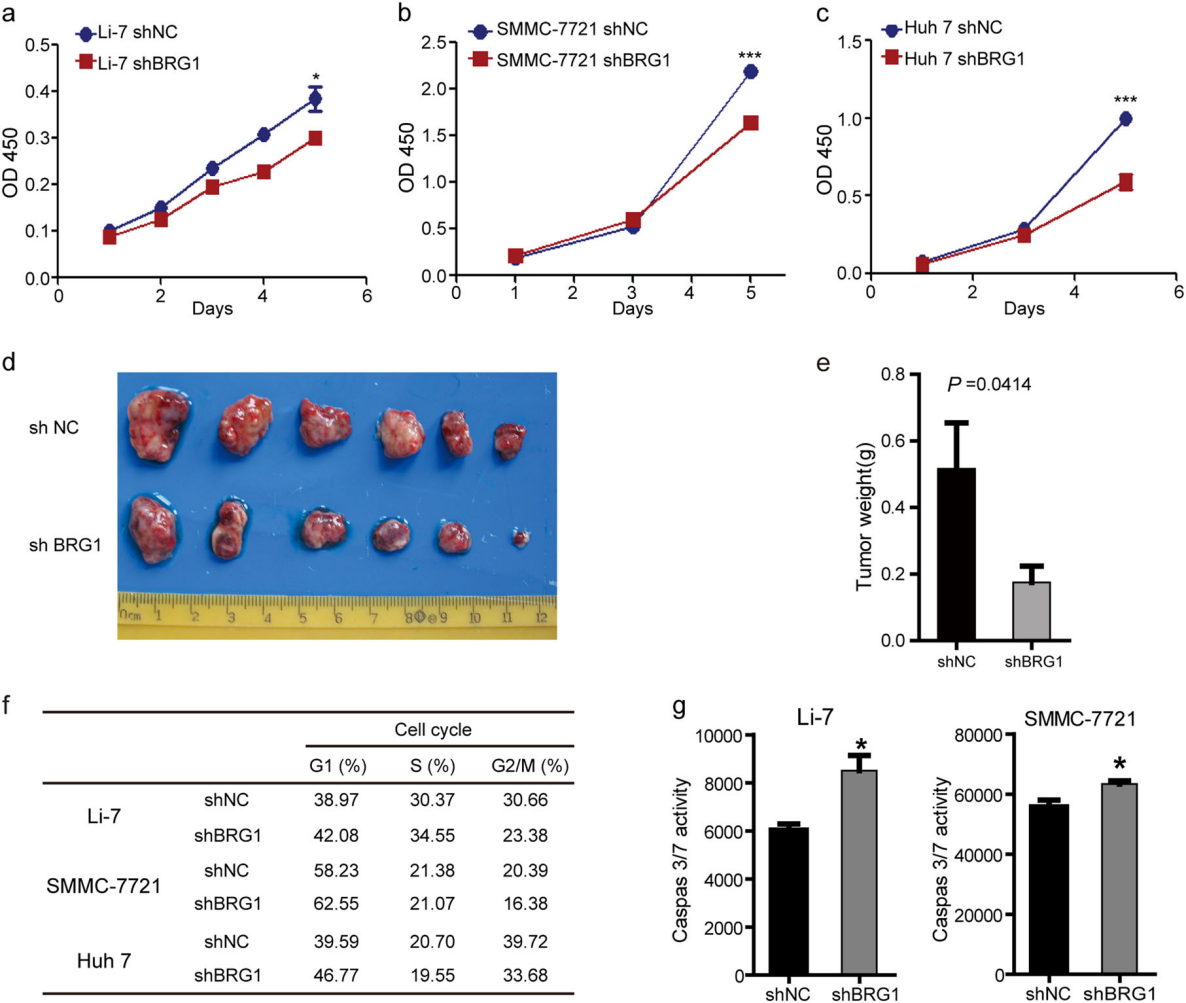
BRG1 knockdown inhibited cell proliferation, arrested cell cycle in G1 phase and induced cell apoptosis **a–c** Representative results of CCK-8 assays assessing the effect of the BRG1 gene on the in vitro proliferation of Li-7, SMMC-7721 and Huh-7 cells after lentivirus-mediated knockdown of BRG1. **d**,** e** The effect of BRG1 on the growth of Huh-7 cells in xenograft nude mice models (*n* = 6) as determined by tumour weight. **f** Statistical analyses of the results of cell cycle analyses. Knockdown of BRG1 arrested the cell cycle in G1/S phase in Li-7, SMMC-7721 and Huh-7 cells. **g** The effect of BRG1 knockdown on apoptosis of both Li-7 and SMMC-7721 cells; the levels of caspase-3/7 were measured using a Caspase-Glo 3/7 assay. All results are shown as the mean ± SEM. *P < 0.05; ***P < 0.001

To investigate the potential functions of BRG1 in hepatic carcinogenesis, we first examined the expression of BRG1 in various HCC cell lines using real-time PCR and western blot analysis. The Huh-7, Li-7 and SMMC-7721 cells, which have a relatively high level of BRG1 expression, were then selected for estimating the effects of stable knockdown of BRG1 using lentiviral infection; both the SK-Hep 1 and MHCC-97H cell lines, which have a very low level of BRG1 expression, were selected for establishing stable BRG1 expressing cell lines (Supplementary Fig. 2). The knockdown efficiencies in Huh-7, Li-7 and SMMC-7721 cells were validated by immunoblotting (Supplementary Fig. 3). Subsequently, the effect of BRG1 knockdown on proliferation was determined with CCK-8 assays. We observed that disruption of BRG1 gene expression inhibited proliferation of HCC cells (Fig. 3a–c). To further characterise the effect of BRG1 on HCC tumour growth in vivo, the flanks of nude mice were subcutaneously inoculated with lenti-shNC and lenti-shBRG1 Huh-7 cells, and these mice were monitored closely for tumour growth for 6 weeks. Importantly, tumour formation in nude mice revealed that knockdown of BRG1 expression significantly reduced tumour size and weight (Fig. 3d and e).

Given that BRG1 is often upregulated in HCC and that knockdown of BRG1 inhibited HCC cell proliferation in vitro and in vivo, we further sought to determine the mechanisms by which BRG1 promoted HCC cell proliferation. Knockdown of BRG1 expression was observed to not only arrest the cell cycle at G1/S but also to promote apoptosis of HCC cells (Fig. 3f and g).

**Fig. 4:**
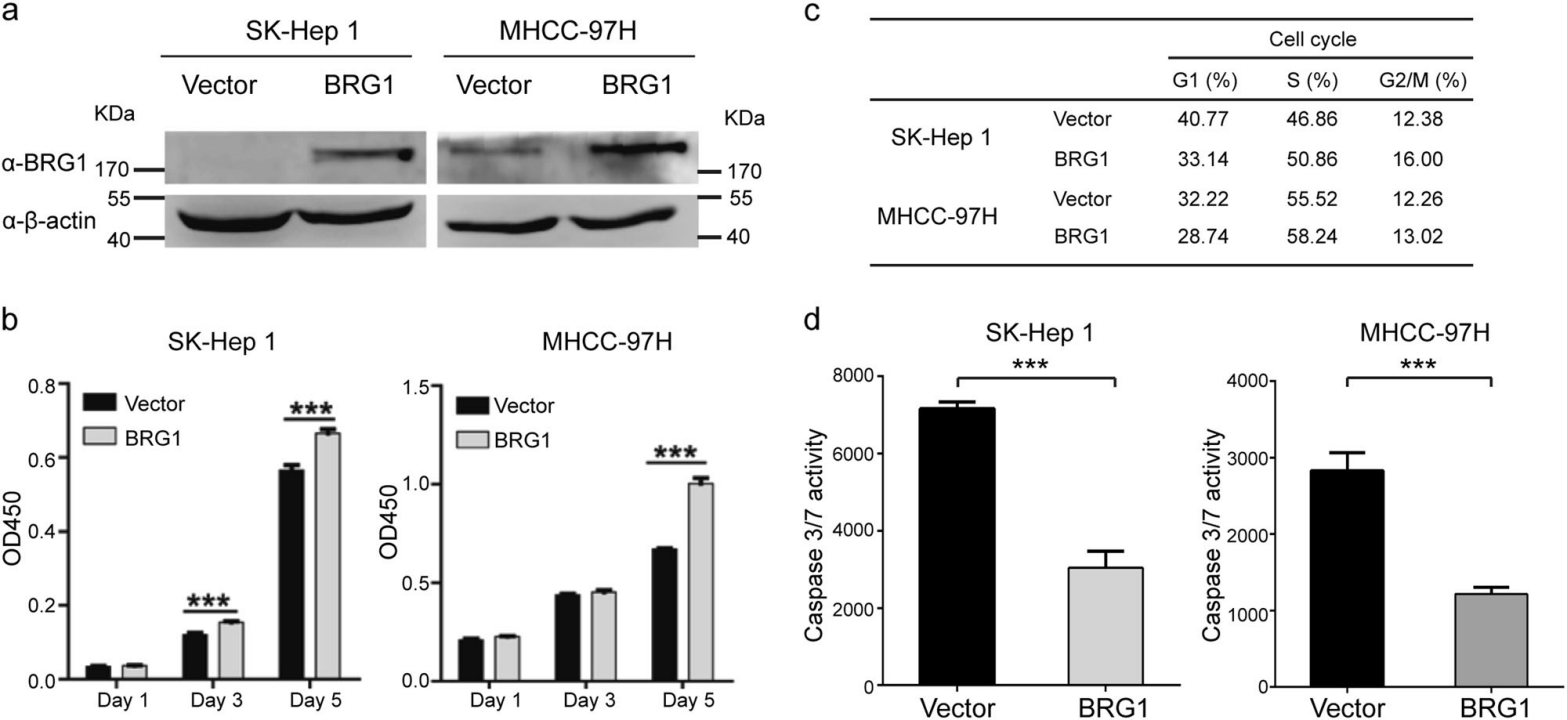
Overexpression of BRG1 promotes cell proliferation **a** The BRG1 protein level was determined with western blotting; β-actin served as a loading control. **b** Representative results of CCK-8 assays assessing the effects of the BRG1 gene on the proliferation of SK-Hep 1 and MHCC-97H cells after lentivirus-mediated overexpression of BRG1. **c** Statistical analyses of the results of cell cycle analyses. Overexpression of BRG1 facilitated S-phase entry in SK-Hep 1 and MHCC-97H cells. **d** The effect of overexpression of BRG1 on apoptosis of both SK-Hep 1 and MHCC-97H cells induced by doxombicin. All results are shown as the mean ± SEM. ***P < 0.001

The BRG1 gene was further overexpressed via lentiviral infection in SK-Hep 1 and MHCC-97H cells, and overexpression was confirmed with immunoblotting (Fig. 4a). Subsequently, we determined the effects of BRG1 overexpression on HCC cell proliferation, cell cycle progression and apoptosis. The results showed that exogenous overexpression of BRG1 increased HCC cell proliferation, facilitated S-phase entry and attenuated caspase 3/7 activity, which was consistent with the above results (Fig. 4b–d). Taken together, these data suggested that BRG1 is a candidate proliferation-promoting factor in HCC and that one mechanism by which BRG1 promotes HCC cell proliferation is regulation of cell cycle progression or apoptotic cell death.

### BRG1 increases SMAD6 expression

**Fig. 5:**
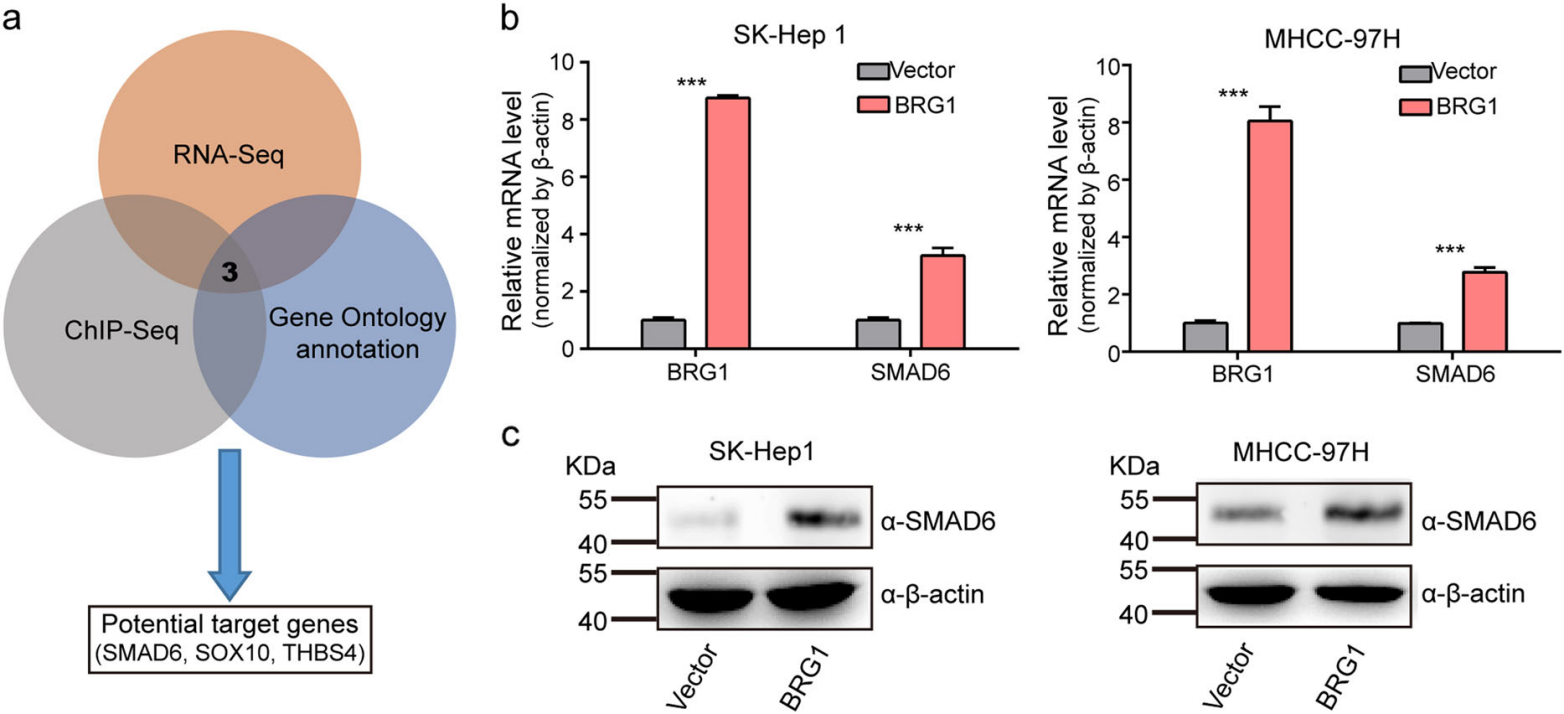
Overexpression of BRG1 increases SMAD6 expression **a** A schematic diagram of the protocol used to search for candidate BRG1 target genes. **b** SMAD6 mRNA level in SK-Hep 1 and MHCC-97H cells with overexpression of BRG1. **c** SMAD6 protein level in SK-Hep 1 and MHCC-97H cells with overexpression of BRG1. β-Actin served as a loading control. All results are shown as the mean ± SEM. ***P < 0.001

As a key component of the SWI/SNF complex, BRG1 may function to control cell proliferation through coordinated regulation of gene expression programmes. To further elucidate the mechanism by which BRG1 modulates the proliferation of HCC cells, we employed two strategies to identify potential downstream targets of BRG1: First, we analysed gene expression profiles induced by BRG1. We overexpressed BRG1 and then performed an RNA-seq analysis in SK-Hep 1 cells to assess potential targets of BRG1. We then mapped changes in transcriptomic profile events compared to control samples and found upregulated genes and downregulated genes (|log_2_-fold change| > 1, Benjamini–Hochberg corrected *P*-value < 0.05). Second, we focused on genes that were associated with ChIP-seq-detected BRG1 binding. Through this combined analysis, a total of 110 candidate targets was found. Of these genes, 68 were upregulated, and 42 were downregulated. Interestingly, after combining the predicted genes and Gene Ontology Biological functional annotation, SMAD6, THBS4 and SOX10 were identified as potential targets of BRG1 (Fig. 5a). To further validate whether these genes are regulated by BRG1, we examined the expression of these genes in BRG1-overexpressing SK-Hep 1 and MHCC-97H cells. We found a significant increase in SMAD6 expression induced by BRG1, whereas SOX10 and THBS4 were not expressed in SK-Hep 1 or MHCC-97H 1 cells (Fig. 5b and c). More importantly, when expression of BRG1 was knocked down in Huh-7 and SMMC-7721 cells, the expression of SMAD6 was decreased compared to control cells transfected with non-targeting siRNA (Supplementary Fig. 4). These results suggest that BRG1 is involved in the regulation of the expression of SMAD6, which may have a functional role in HCC progression.

### BRG1 promotes cell proliferation through upregulation of SMAD6

**Fig. 6:**
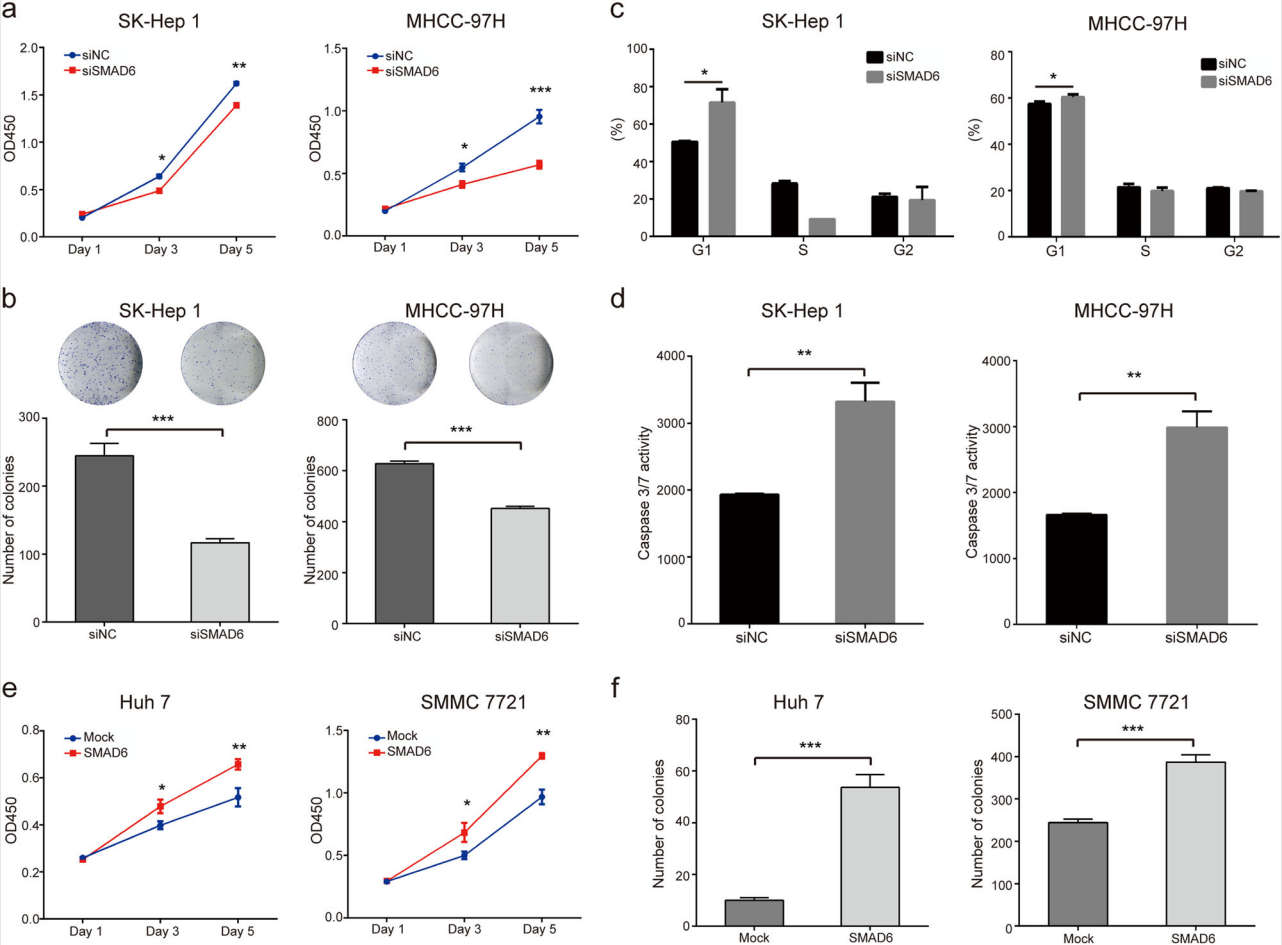
SMAD6 knockdown inhibited cell proliferation, arrested cell cycle in G1 phase and induced apoptosis of HCC cells **a**,**b** Representative results of CCK-8 assays and colony formation assays showing the effect of knockdown of SMAD6 gene expression on the proliferation of SK-Hep 1 and MHCC-97H cells. **c** Statistical analyses of the results of cell cycle analyses. Knockdown of SMAD6 arrested the cell cycle in G1/S phase in SK-Hep 1 and MHCC-97H cells. **d** The effect of SMAD6 knockdown on apoptosis of both SK-Hep 1 and MHCC-97H cells. **e**,**f** Representative results of CCK-8 assays and colony formation assays showing the effect of lentivirus-mediated overexpression of SMAD6 on proliferation of Huh-7 and SMMC-7721 cells. All results are shown as the mean ± SEM. *P < 0.05; **P < 0.01; ***P < 0.001

To determine the functional roles of SMAD6 in HCC, we first knocked down the expression of the SMAD6 gene in SK-Hep1 and MHCC-97H cells. The knockdown efficiency was validated by western blotting (Supplementary Fig. 5a). Subsequently, the effect of SMAD6 knockdown on proliferation was determined with CCK-8 assays and colony formation assays. Our results showed that disruption of SMAD6 gene expression significantly decreased the proliferation of SK-Hep1 and MHCC-97H cells (Fig. 6a and b). In addition, we found that knockdown of SMAD6 expression not only arrested the cell cycle at the G1/S phase but also promoted apoptosis of HCC cells. These results are similar to the results observed when BRG1 expression was knocked down (Fig. 6c and d).

Furthermore, we overexpressed the SMAD6 gene in Huh-7 and SMMC-7721 cells using a lentivirus system to establish two stable SMAD6-overexpressing cell lines. The overexpression efficiency was determined by western blotting (Supplementary Fig. 5b). The results showed that overexpression of the SMAD6 gene significantly increased the proliferation of Huh-7 and SMMC-7721 cells (Fig. 6e and f). The above results indicate that SMAD6 significantly promotes HCC cell proliferation.

**Fig. 7:**
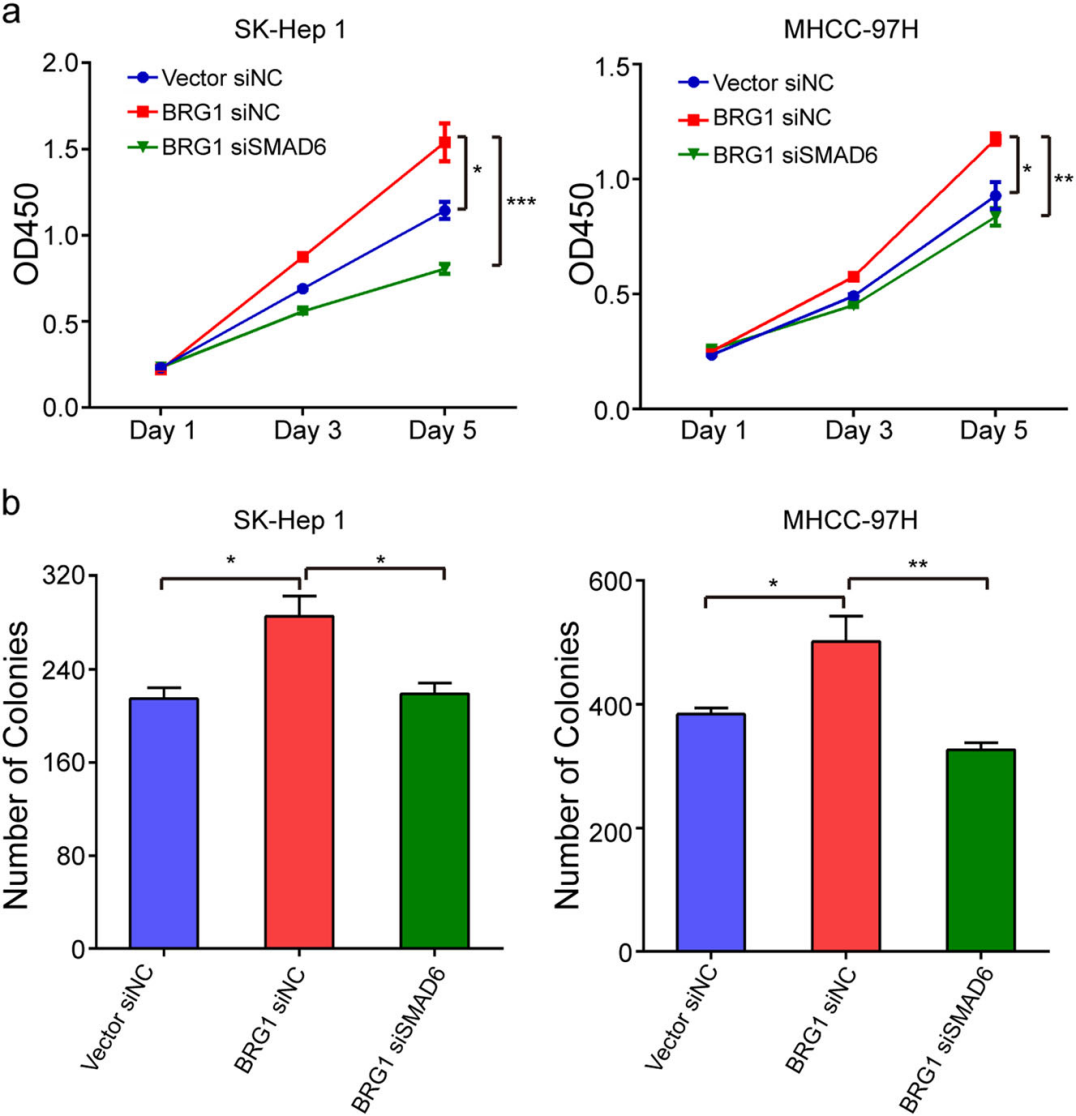
BRG1 promotes cell proliferation through upregulation of SMAD6 **a**,**b** Representative results of CCK-8 assays and colony formation assays of SK-Hep 1/MHCC-97H-Mock cells or cells stably expressing BRG1 transfected with small interfering RNA (siRNA) against SMAD6 or with a negative control (NC). *P < 0.05; **P < 0.01; ***P < 0.001; *OD* optical density

Since SMAD6 significantly promoted HCC cell proliferation and BRG1 upregulated the expression of SMAD6, we aimed to determine whether BRG1 promoted liver cancer cell proliferation through SMAD6. Inhibiting SMAD6 expression abrogated the increase in cell proliferation induced by BRG1 (Fig. 7). These results indicate that BRG1 promotes cell proliferation through upregulation of SMAD6.

### BRG1 upregulates expression of SMAD6 through HIC2 and NR4A2

**Fig. 8:**
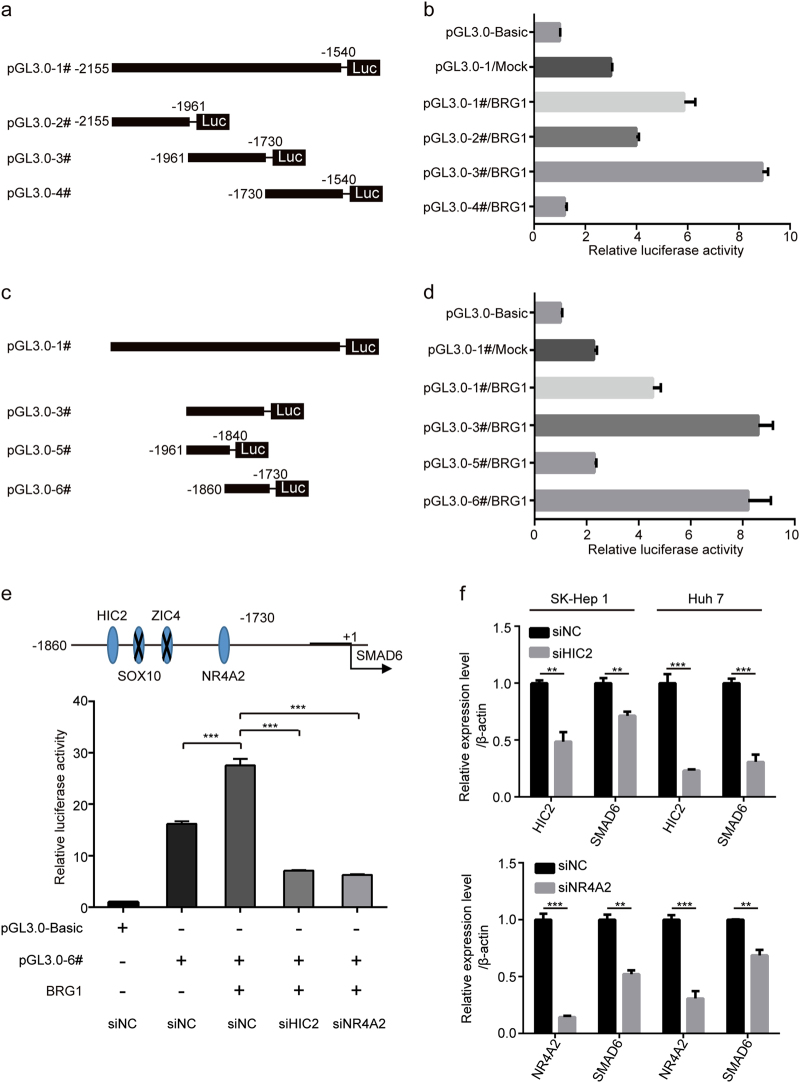
SMAD6 expression is regulated by BRG1 **a** Schematic representation of human SMAD6 promoter reporter constructs. Fragments of various lengths between −2155 and −1540 bp of SMAD6 were cloned downstream of the firefly luciferase reporter. **b** Luciferase activity in HEK293T cells transfected with firefly luciferase reporter plasmids containing various upstream regions of SMAD6. A Renilla luciferase reporter was cotransfected with a pGL3-basic or plasmid reporter. **c** Fragments of various lengths between −1961 and −1730 bp of SMAD6 were cloned downstream of the firefly luciferase reporter. **d** Luciferase activity in HEK293T cells transfected with firefly luciferase reporter plasmids containing various upstream regions of SMAD6. **e** Putative transcription factor binding sites in the region between −1860 and −1730 bp upstream of SMAD6 (upper panel). Luciferase activity associated with the region between −1860 and −1730 bp of SMAD6 in HEK293T cells transfected with small interfering RNA (siRNA) against HIC2, NR4A2 or with a negative control (NC) (lower panel). **f** HIC and SMAD6 expression in SK-Hep 1 and Huh-7 cells after HIC2 was knocked down as assessed with real-time PCR (upper panel). NR4A2 and SMAD6 expression in SK-Hep 1 and Huh-7 cells after NR4A22 was knocked down (lower panel). Data represent the mean ± SEM. **P < 0.01; ***P < 0.001

BRG1 is a part of the large ATP-dependent chromatin remodelling complex SWI/SNF, which is capable of mobilising nucleosomes, translocating DNA and DNA loop formation that can generate sites that are more accessible to DNA-binding factors. Thus, DNA-binding factors, such as transcription factors, are likely responsible for the upregulation of SMAD6 in HCC. According to the results of ChIP-seq, we found that a site −1846 bp upstream of SMAD6 was the peak centre for binding of BRG1. A series of luciferase reporter constructs containing fragments from this extended region (from −2155 to −1540 bp) were cloned into a pGL3-basic vector (Fig. 8a), and luciferase activity was measured after transfection of these constructs and BRG1/Mock into SK-Hep 1 cells. The highest activity was associated with the −1860 to −1730 bp fragment (Fig. 8b–d), indicating that it contained the regulatory elements critical for the transcription of SMAD6 induced by BRG1.

Next, we predicted the transcription factors that were likely to bind to the key upstream region (−1860 to −1730 bp) of the SMAD6 gene using the JASPAR database. The transcription factors included HIC2, SOX10, ZIC4 and NR4A2. We transfected HEK293T and SK-Hep 1 cells with siRNA against the four transcription factors. At 48 h after transfection, RNA was collected and reverse transcribed into cDNA. The interference efficiency of each siRNA was determined. The SOX10 and ZIC4 genes were not expressed in HEK293T and SK-Hep 1 cells, but the expression of the other two genes was significantly reduced after siRNA-mediated interference (Supplementary Fig. 6). Furthermore, HEK293T cells were transiently co-transfected with the siRNAs against the two genes, the luciferase reporter vector containing the region between −1860 and −1730 bp, and pWPXL-BRG1/Mock plasmids. Subsequently, the changes in luciferase activity were examined. The results showed that overexpression of BRG1 failed to induce the corresponding changes in luciferase activity after siRNA-mediated downregulation of HIC2 and NR4A2 (Fig. 8e). In addition, BRG1-mediated upregulation of SMAD6 expression was not observed after interference with HIC2 and NR4A2 expression (Fig. 8f). The above results indicate that the transcription factors HIC2 and NR4A2 might affect BRG1-regulated SMAD6 expression.

## Discussion

In this study, we first investigated the expression of BRG1 in 140 paired HCC tissues and adjacent NTs, and we demonstrated that BRG1 was upregulated in HCC. Consistent with our findings, analysis of BRG1 expression in TCGA and Oncomine datasets confirmed higher BRG1 expression in HCC tissues than in normal liver tissues in two independent cohorts of HCC specimens. Importantly, in accordance with the immunohistological results, high BRG1 expression in the nucleus was a frequent event in human HCC tissues and was correlated with serum AFP concentration. These results suggest that BRG1 may play an important role in HCC.

Although several advances in HCC prevention, early detection, and diagnosis have been efficacious and have reduced the incidence and mortality of HCC, the prognosis of patients with HCC is still unsatisfactory. The 5-year risk of recurrence of HCC after resection is as high as 70% because the underlying chronic liver disease continues to put the patient at risk for the development of new HCC^17,18^. Improving the survival rate of patients with HCC requires that clinicians engage in active treatment of recurrence. In this study, we identified BRG1 for predicting early recurrence based on protein expression data that was obtained via immunohistochemistry (IHC) from samples taken from HCC patients who all relapsed a few years later. By combining these results with the findings of previous reports, we propose that simultaneous analysis of multiple genes, including nuclear expression of BRG1, may represent useful biomarkers for the risk of HCC recurrence and may be used to identify patients who should be closely monitored after curative HCC ablation.

BRG1 is a part of the large ATP-dependent chromatin remodelling complex SNF/SWI. SWI/SNF chromatin remodelling complexes use the energy of ATP hydrolysis to remodel nucleosomes and are capable of mobilizing nucleosomes both by sliding and by catalysing the ejection and insertion of histone octamers to generate sites that are more accessible to DNA-binding factors^19^. The SWI/SNF chromatin-remodelling complex plays essential roles in a variety of cellular processes including differentiation, proliferation and DNA repair. Loss of SWI/SNF subunits has been reported in several malignant cell lines and tumours, and a large number of experimental observations suggest that this complex functions as a tumour suppressor^9^. BRG1 inactivating mutations, with a corresponding loss of protein, have been identified in a variety of cell lines and primary tumours, such as pancreatic, breast and prostate cancer cell lines and in lung cancer and medulloblastoma^10^. Conversely, the expression of BRG1 was frequently elevated in melanoma and colorectal carcinoma specimens. BRG1 plays an important role in melanoma metastasis through target genes^20^ and the process of colorectal cancer development by activating the PI3K-Akt signalling pathway and resultant upregulation of cyclin D1 levels^21^. In human glioma, BRG1 expression is increased and knockdown of BRG1 inhibits cell growth due to the G1 phase arrest by downregulating cyclin D1^22^. Although the function of BRG1 in various cancers is ambiguous, regulation of the expression of BRG1 is tissue specific, and epigenetic modifications, as well as potential DNA-binding proteins may be involved in the function of BRG1. DNA sequencing and mutation analysis in HCC identified significantly mutated genes, including BRG1, which exhibited near significance by MutSigCV analysis^23^. Recently, Zhu et al. showed that BRG1 was highly expressed in HCC tumours, and increased BRG1 levels was positively correlated with severity of HCC patients^24^. In this study, we demonstrate that BRG1 promotes HCC cell proliferation in vitro and in vivo. Moreover, we observed that BRG1 not only facilitates S-phase entry but also attenuates cell apoptosis. So to our knowledge, BRG1 may act as an oncogene in HCC.

To further explore the underlying mechanism by which BRG1 promotes cell proliferation, we overexpressed BRG1 and performed ChIP-seq combined with RNA-seq analysis, as well as quantitative real-time PCR and demonstrated that overexpression of BRG1 upregulates SMAD6 expression. The SMAD family of proteins transduce signals from the TGF-ß superfamily ligands that regulate cell proliferation, differentiation and death through activation of receptor serine/threonine kinases. SMAD6 is an inhibitory SMAD that competitively binds type I receptors or SMAD4 to inhibit SMAD1/5/8 phosphorylation or nuclear translocation, respectively, and primarily inhibits bone morphogenetic protein (BMP) signalling^25–27^. The functional linkage between SMAD6 and cancer most likely impacts cancer initiation and progression, because SMAD6 expression is deregulated in cancers. In NSCLC, increased SMAD6 expression is linked with poor prognosis^28^. Furthermore, increased SMAD6 expression contributes to a malignant phenotype, which is apparent by the reported dramatic increase in anchorage-independent growth of pancreatic ductal cancer cells^29^. Here, we observed that SMAD6 promoted cell proliferation in HCC and BRG1 promoted tumour cell proliferation by upregulating the expression of SMAD6, at least partially. These results indicated that BRG1 regulates the expression of SMAD6. In addition, the transcription factors HIC2 and NR4A2 may be involved in the upregulation of SMAD6 induced by BRG1. However, further investigation is required to elucidate the detailed mechanisms.

In conclusion, our findings provide mechanistic insight into how the SWI/SNF subunit BRG1 promotes cell proliferation. The crucial role of BRG1-induced SMAD6 was thereby elucidated. Importantly, nuclear expression of BRG1 may be a useful biomarker of the risk of HCC recurrence. This information will be valuable for determining prognoses and treating HCC patients.

## Materials and methods

### Patients and specimens

This study involved three independent cohorts of patients with HCC: cohort 1, comprising 140 patients with HCC from Qi Dong Liver Cancer Institute (Jiangsu, PR China); cohort 2 and cohort 3, comprising 129 and 118 HCC patients, respectively, from the Eastern Hepatobiliary Surgery Hospital (Shanghai, PR China). We obtained snap-frozen HCC tissues and adjacent NTs with HCC who had first undergone radical resection of HCC. Tissues from cohort 1 were collected from patients between 2000 and 2005. Tissues from cohort 2 were collected from patients between January and December 2000. Patients with HCC from cohort 3 had undergone radical resection of HCC, suffered a relapse a few years later, and then underwent a second resection of HCC. The patients’ clinicopathological information including age at diagnosis, hepatitis history, level of α-Fetoprotein, liver cirrhosis, tumour size, tumour number, vascular invasion and TNM stage was obtained from the archive of the pathology department. The follow-up procedures and postoperative treatments were based on a uniform guideline and have been described previously^30^. Tumour differentiation was graded using the Edmondson grading system. Clinical staging was performed according to the 6th edition of the AJCC/UICC TNM classification system. The time to recurrence and OS were calculated from the date of surgery to the date of the first recurrence and death, respectively. The data were censored at the last follow-up for patients without relapse or upon death. Institutional review board approval was obtained, and each patient provided written informed consent.

### Antibodies, plasmids and reagents

Anti-BRG1 and anti-SMAD6 antibodies were purchased from Santa Cruz Biotechnology (Texas, CA, USA); anti-β-actin was obtained from Sigma (St. Louis, MO, USA); anti-GAPDH was obtained from KangChen Bio-tech (Shanghai, PR China). Lentiviral shRNA vectors targeting BRG1 and scrambled control shRNA vectors were purchased from Open Biosystems (Thermo scientific). siRNA targeting SMAD6 and negative control siRNA were ordered from RiboBio (Guangzhou, PR China).

### Cell culture

Eight liver cancer cell lines were used in this study: SNU-449, HCC-LM3, Hep 3B, Li-7, Huh-7, MHCC-97H, SMMC-7721 (Shanghai Second Military Medical University, Shanghai, PR China) and SK-Hep1 (ATCC, HTB-52). The SMMC-7721 cells were maintained in Dulbecco’s modified Eagle medium (DMEM) (Invitrogen) supplemented with 10% newborn calf serum (Invitrogen, Carlsbad, CA, USA). The other seven cancer cell lines and HEK293T cells were cultured in DMEM supplemented with 10% foetal bovine serum. All cells were cultured at 37 °C with 5% CO_2_.

### Transient transfection and RNA interference

Cells were transfected using Lipofectamine 2000 reagent (Invitrogen) according to the manufacturer’s instructions. The total amount of transfected plasmid DNA was held constant between experimental conditions by adding of an empty-vector plasmid. For small interfering RNA (siRNA) transfection, cells seeded in antibiotic-free medium at 30–50% confluence were transfected with 50 nM siRNA duplexes using RNAiMAX (Invitrogen) according to the manufacturer’s instructions.

### Lentiviral vector construction, packaging and infection

The entire coding sequence of target cDNA was amplified and cloned into a pWPXL vector. Virus particles were harvested 48 h after transfection of HEK293T cells with the pWPXL-vector or indicated plasmid along with the packaging plasmid psPAX2 and the VSV-G envelope plasmid pMD2.G (gifts from Didier Trono) using Lipofectamine 2000 reagent (Invitrogen, Carlsbad, CA).

### Quantitative real-time PCR analysis

Total RNA was extracted from tissues or cells using TRIzol reagent (Invitrogen) according to the manufacturer’s instructions. Reverse-transcription PCR (RT-PCR) was performed with a Prime-Script RT Reagent Kit (TaKaRa, Dalian, China). Gene expression levels were determined with quantitative real-time PCRs and normalised against endogenous β-actin as a control using SYBR Premix Ex Taq (TaKaRa). Data were analysed using a ΔΔCt approach and are expressed as the target gene/β-actin ratio [2^−ΔCt(target gene-β-actin)^].

### Western blot analysis

Cells were harvested by scraping them into an SDS sample buffer containing a cocktail of protease inhibitors (Pierce, Rockford, IL, USA). Similar amounts of proteins were loaded into a gel, separated by SDS-PAGE, and transferred to a nitrocellulose membrane (Bio-Rad, Hercules, CA, USA). The membrane was blocked with TBST (0.05% Tween 20 in TBS) containing 5% skim milk and then incubated overnight with the indicated antibodies at 4 °C. The membrane was washed three times in TBST and then incubated with an HRP-conjugated secondary antibody (Pierce) (1:2000) for 2 h at room temperature. The immunocomplexes were detected using enhanced chemiluminescence (Pierce).

### Cell proliferation and colony formation assays

Cell proliferation was monitored by counting viable cells using Cell Counting Kit (CCK)-8 (Dojindo, Kumamoto, Japan). Cells were seeded at 1000 cells/well in 96-well plates and incubated. Then, 10 μl of the CCK-8 solution was added to the triplicate wells and incubated for 2 h. Subsequently, the absorbance at 450 nm was measured to calculate the number of viable cells in each well.

For colony formation assays, cells were trypsinized, resuspended, seeded into six-well plates at a concentration of 500 cells per well, and cultured at 37 °C for 1–2 weeks. The medium was changed every 3–4 days. At the end of the incubation, the cells were fixed with 100% methanol and stained with 0.1% crystal violet. Megascopic cell colonies were counted using Image-Pro Plus 5.0 (Media Cybernetics, Bethesda, MD).

### Xenograft experiments

Xenograft experiments were performed as described previously. Briefly, Huh-7-shNC cells and Huh-7-shBRG1 cells with stable knockdown of BRG1 (2 × 10^6^ per mouse) were injected subcutaneously into the right upper flank region of nude mice. The mice were monitored weekly for tumour size and evidence of morbidity. Approximately 6 weeks after injection, the tumours were dissected and weighed. Mouse experiments were conducted in accordance with the Guide for the Care and Use of Laboratory Animals of Fudan University. The protocol was approved by the Committee on the Ethics and Welfare of Laboratory Animal Science of Fudan University.

### Tissue microarray and IHC

A tissue microarray was constructed as described previously^30^. Core samples were obtained from representative regions of each tumour based on haematoxylin and eosin staining. Duplicate 1-mm cores were taken from different areas of the same tissue block for each case (intratumoral tissue and peritumoral tissue). Serial sections (4 μm thick) were placed on slides coated with 3-aminopropyltriethoxysilane. An IHC analysis was performed as described previously. All samples were anonymously and independently scored by two investigators. In the case of disagreement, the slides were re-examined, and a consensus was reached by the observers.

### Cell cycle analysis and apoptosis assays

Cells were fixed in 70% ethanol at –20 °C for 24 h, stained with 50 μg/ml propidium iodide (Kaiji, NanJing, China), and analysed using a FACSCaliber flow cytometer (BD Bioscience, MA). The results were analysed using ModFit software (BD Bioscience). For apoptosis quantification, cellular apoptosis was determined by measurement of caspase 3 and 7 activity using a luminometric Caspase-Glo 3/7 assay (Promega) according to the manufacturer’s protocol.

### Statistical analysis

The experiments were repeated at least three times, and the results are expressed as the mean and the standard error of the mean (SEM). Unless otherwise noted, Student’s *t*-test (two-tailed) and one-way analysis of variance followed by Dunnett multiple comparisons test were used to compare the differences between two groups and more than two groups, respectively. A chi-squared (*χ*^2^) test was used to evaluate the association between BRG1 expression and clinicopathological parameters. The cumulative recurrence and survival rates were determined using the Kaplan–Meier method (log-rank test). A *P*-value of <0.05 was considered significant. All statistical analyses were performed using IBM SPSS Statistics V19 package (Armonk, NY, USA).

## Electronic supplementary material

Supplementary figures
