# Supplementary material for: Hepatic SMARCA4 predicts HCC recurrence and promotes tumour cell proliferation by regulating SMAD6 expression
Source: Cell Death Dis. 2018 Jan 19;9(2):59. doi: 10.1038/s41419-017-0090-8 (PMC5833410; doi:10.1038/s41419-017-0090-8)
Supplement: Supplementary file 1 — Supplementary figures [file 41419_2017_90_MOESM1_ESM.docx]

**Hepatic SMARCA4 Predicts HCC Recurrence and Promotes Tumour Cell Proliferation by Regulating SMAD6 Expression**

Zhiao Chen^1,*^, Xinyuan Lu^2,*^, Deshui Jia^1,*^, Ying Jing^3^, Di Chen^1^, Qifeng Wang^1^, Fangyu Zhao^3^, Jinjun Li^3^, Ming Yao^3^, Wenming Cong^2^, Xianghuo He^1,†^

1. Fudan University Shanghai Cancer Center and Institutes of Biomedical Sciences; Department of Oncology, Shanghai Medical College, Fudan University, Shanghai 200032, China;

2. Department of Pathology, Eastern Hepatobiliary Surgery Hospital, Second Military Medical University, Shanghai 200438, China;

3. State Key Laboratory of Oncogenes and Related Genes, Shanghai Cancer Institute, Renji Hospital, Shanghai Jiao Tong University School of Medicine, Shanghai 200032, China;

^*^These authors contributed equally to this work.

***Corresponding authors**

**Address:** Fudan University Shanghai Cancer Center and Institutes of Biomedical Sciences, Fudan University, Shanghai 200032, China. Tel./fax: +86 21 34777577. Email address: [xhhe@fudan.edu.cn](mailto:xhhe@fudan.edu.cn).

**Supplementary figure 1**


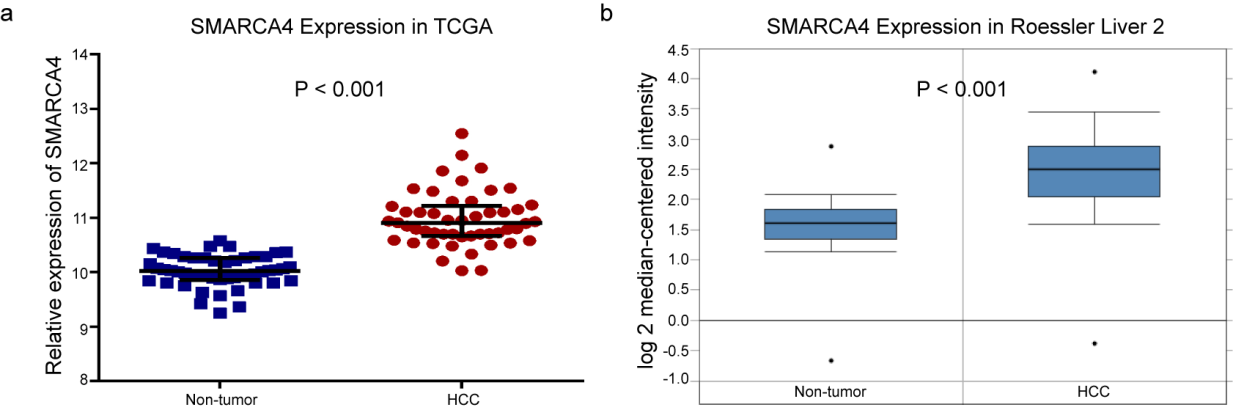


**Figure S1 The expression of BRG1 was upregulated in HCC**

The expression levels of BRG1 in 50 tumour and adjacent normal tissues from TCGA and 445 samples from Roessler Liver 2 in the Oncomine dataset.

**Supplementary figure 2**


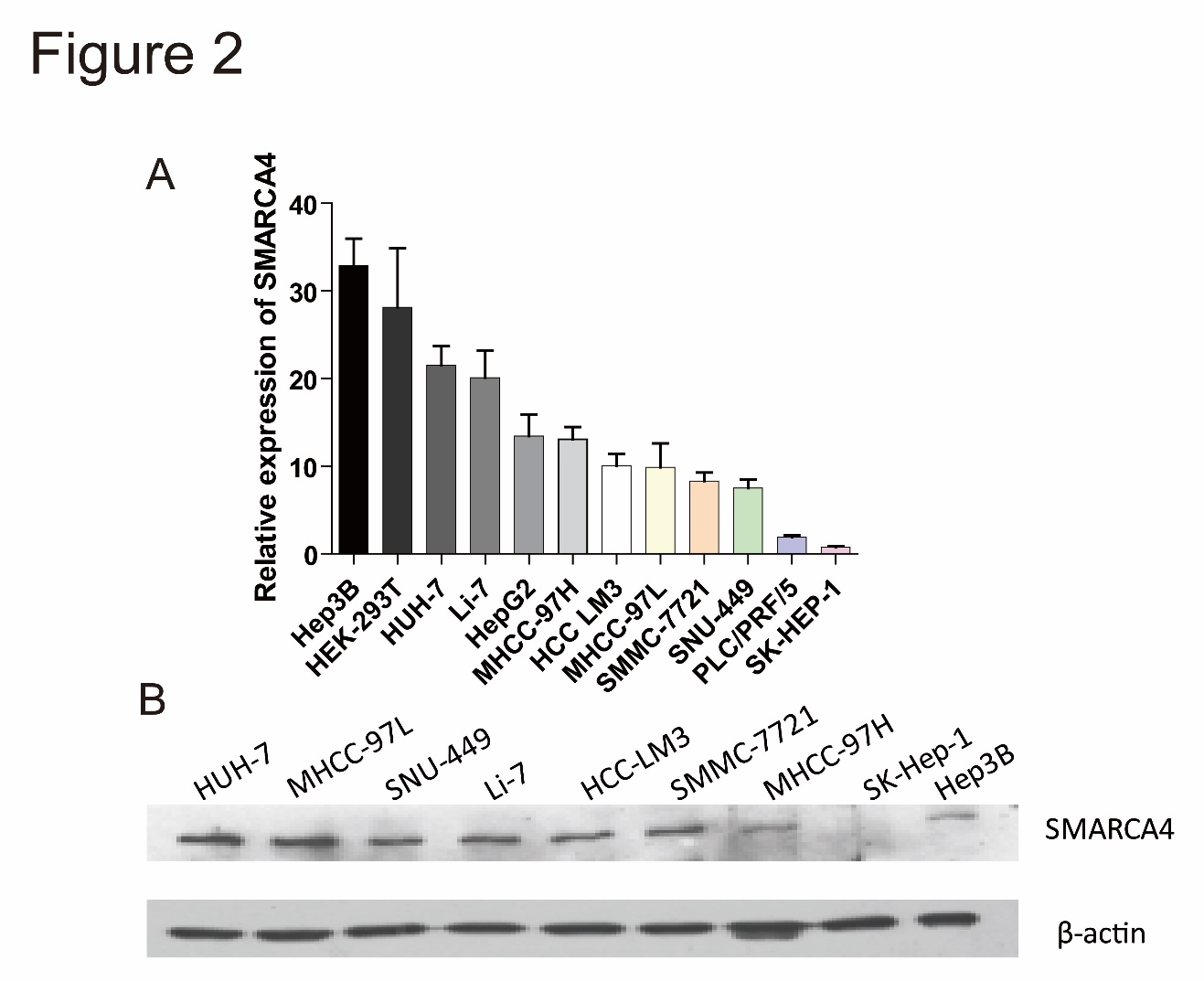


KDa

55

40

170

**Figure S2 BRG1 expression level in HCC cell lines**

**(a, b)** Expression levels of SMARCA4/BRG1 mRNA (a) and protein (b) in liver cancer cells. Cell lysates were examined using western blotting with equal amounts of protein. β-Actin served as a loading control.

**Supplementary figure 3**


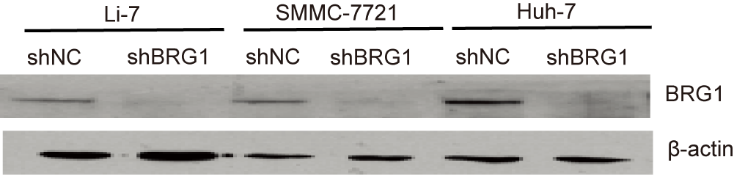


KDa

40

55

170

**Figure S3 knockdown of BRG1 expression in HCC cell lines**

The BRG1 protein level in Li-7, SMMC-7721, and Huh-7 cells transfected with shRNA against BRG1. β-Actin served as a loading control.

**Supplementary figure 4**


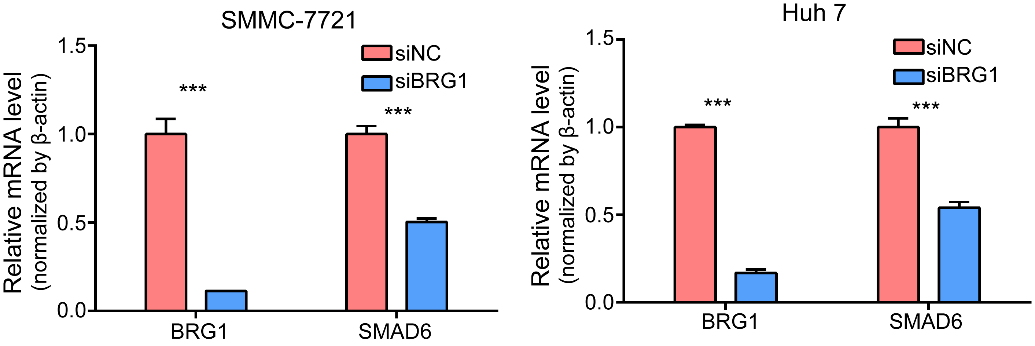


**Figure S4 Knockdown of BRG1 reduced SMAD6 expression**

The SMAD6 mRNA level in BRG1-overexpressing SMMC-7721 and Huh-7 cells. All results are shown as the mean ± SEM. ***P < 0.001.

**Supplementary figure 5**

**
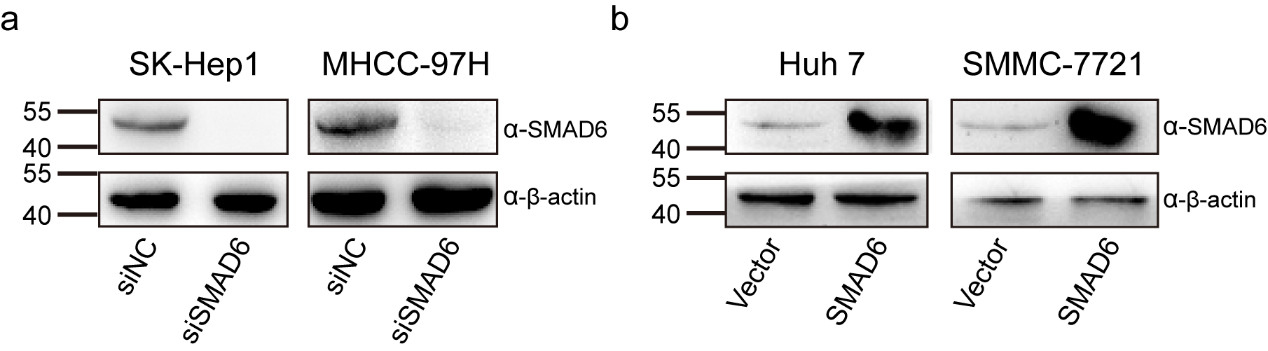
**

**Figure S5 Knockdown and overexpression of SMAD6 expression in HCC cell lines**

**(a)** The SMAD6 protein level in SK-Hep 1 and MHCC-97H cells transfected with small interfering RNA (siRNA) against SMAD6. **(b)** The SMAD6 protein level in SMAD6-overexpressing SMMC-7721 and Huh-7 cells. β-Actin served as a loading control.

**Supplementary figure 6**


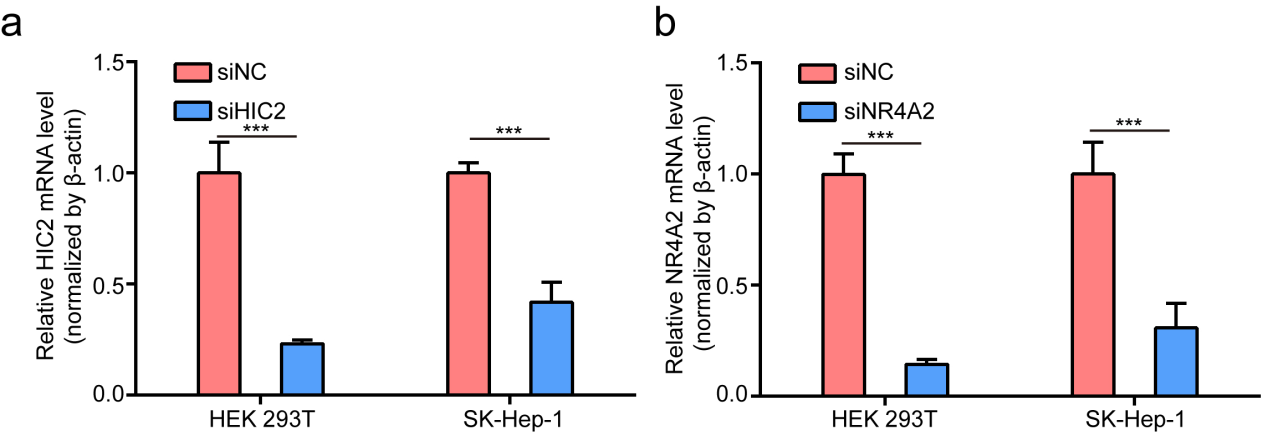


**Figure S6 The knockdown efficiencies of HIC2 and NR4A2 measured by q-PCR in HEK 293T and SK-Hep-1 cells**

(a) The HIC2 mRNA level in HEK293T and SK-Hep 1 cells with knockdown of HIC2. (b) The NR4A2 mRNA level in HEK293T and SK-Hep 1 cells with knockdown of NR4A2. All results are shown as the mean ± SEM. ***P < 0.001.
